# Sedation in the ICU: nurses' perceptions of practices and influencing factors

**DOI:** 10.1186/cc10931

**Published:** 2012-03-20

**Authors:** B Sneyers, PF Laterre, MM Perreault, A Spinewine

**Affiliations:** 1 Université catholique de Louvain, Louvain Drug Research Institute, Brussels, Belgium; 2 Université catholique de Louvain, Cliniques Universitaires St-Luc, Brussels, Belgium; 3 Université de Montreal, Canada

## Introduction

Our goals are to describe adherence to sedation recommendations [1] in Belgian ICUs and to identify major factors influencing practices.

## Methods

A national survey including all nurses working in Belgian ICUs was conducted with seven nurses sampled per hospital. A validated self-administered paper survey was designed based on a literature review and data from a previous qualitative study. Topics addressed were current practices and reasons for (non)compliance to sedation recommendations such as use of sedation scales and daily sedation interruption (DSI). Four postal reminders were sent.

## Results

The response rate was 70% (*n *= 587/840 nurses from 99/120 hospitals). Sedation scales are available to 89% of nurses and frequency of use is variable (≤1×/day: 13%, 3 to 4×/day: 31%, ≥6×/day: 56%). When sedation scales are available, perceived indications are monitoring of sedation and analgesia (96% and 31% of nurses respectively) and dosing adjustment for sedatives and analgesics (14% and 28% of nurses respectively). DSI is infrequently used (never used: 38% of respondents, used for <25% of patients: 47% of respondents, used for 25 to 75% of patients: 12% of respondents, used for >75% of patients: 3% of respondents). Numerous barriers for wide implementation are identified, mainly lack of outcome expectancy, as DSI is perceived to impair patient outcomes. It is perceived that DSI increases the risk of complications such as unplanned extubation and pulling of lines and tubes (79% of nurses agree), impairs patients' comfort (59% of nurses agree), and creates traumatic memories in the intubated patients (36% of nurses agree). Moreover, 63% of nurses agree that they would prefer no DSI if they were an intubated patient. Other barriers are related to knowledge, as 26% of nurses do not know the practice, and to behaviour, as 53% of respondents feel DSI is difficult to implement because of organizational constraints.

## Conclusion

Sedation scales are widely used in Belgium, while use of DSI is low. Barriers impairing adherence to recommendations were identified. Perception that sedation scales are not used for sedative dosing adjustments is present, as well as inadequate use for analgesia. Fear of worsening patient outcomes using DSI is present, contrasting with current literature. A similar survey addressing physicians' perceptions is ongoing.
